# Non-canonical amino acids as a tool for the thermal stabilization of enzymes

**DOI:** 10.1093/protein/gzad003

**Published:** 2023-03-10

**Authors:** Tim Lugtenburg, Alejandro Gran-Scheuch, Ivana Drienovská

**Affiliations:** Department of Chemistry & Pharmaceutical Sciences, Vrije Universiteit Amsterdam, 1081 HZ Amsterdam, The Netherlands; Department of Chemistry & Pharmaceutical Sciences, Vrije Universiteit Amsterdam, 1081 HZ Amsterdam, The Netherlands; Department of Chemistry & Pharmaceutical Sciences, Vrije Universiteit Amsterdam, 1081 HZ Amsterdam, The Netherlands

**Keywords:** biocatalysis, enzyme stability, halogenated ncAAs, ncAAs, selective pressure incorporation, stop codon suppression, unnatural amino acid

## Abstract

Biocatalysis has become a powerful alternative for green chemistry. Expanding the range of amino acids used in protein biosynthesis can improve industrially appealing properties such as enantioselectivity, activity and stability. This review will specifically delve into the thermal stability improvements that non-canonical amino acids (ncAAs) can confer to enzymes. Methods to achieve this end, such as the use of halogenated ncAAs, selective immobilization and rational design, will be discussed. Additionally, specific enzyme design considerations using ncAAs are discussed along with the benefits and limitations of the various approaches available to enhance the thermal stability of enzymes.

## Graphical abstract

**Figure:**
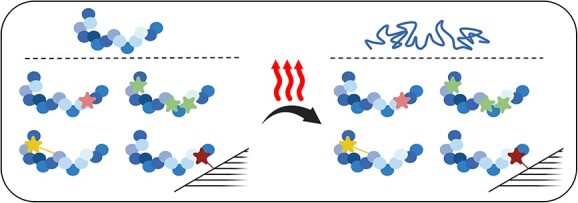

## Introduction

Enzymes are attractive catalysts for the pharmaceutical, biotechnological and chemical industries due to their ability to catalyze reactions with exquisite performance. A major advantage is their remarkable chemo-, regio- and enantioselectivity (Sheldon and Woodley, 2018; Fürst *et al.*, 2019; Bell *et al.*, 2021; Winkler *et al.*, 2021; Yi *et al.*, 2021; Hanefeld *et al.*, 2022). Moreover, these biocatalysts fulfill the principles of green chemistry. As a consequence, biocatalysis is a maturing field, becoming a green alternative to conventional chemical methods, which tend to be less eco-friendly (Sheldon and Woodley, 2018). Despite advancements in the field, the limited ability of enzymes to maintain stability at high temperatures remains a significant challenge for industrial applications. This is primarily due to the fact that many biotechnologically useful enzymes have been sourced from mesophilic organisms that have evolved to have relatively low thermal stability. However, stabilized enzymes are attractive to perform chemical reactions at higher temperatures, as it allows for higher substrate solubility, reduced microbial contamination and often a more favorable equilibrium (Bommarius and Paye, 2013). The use of thermostable enzymes has various practical applications with the widespread use of thermostable deoxyribonucleic acid (DNA) polymerases in molecular biology techniques as a notable example (Rigoldi *et al.*, 2018). Other applications include enzymatic carbon dioxide capture, starch conversion in the food and beverage industry, enzymatic bio-pulping for the paper industry or the synthesis of fine and bulk molecules for pharmaceutical and chemical products (Koch *et al.*, 1990; Sharma and Satyanarayana, 2012; Han *et al.*, 2013; Satyanarayana *et al.*, 2013; Sarmiento *et al.*, 2015; Rigoldi *et al.*, 2018). Furthermore, increased thermostability has been reported to improve tolerance toward co-solvents and mutational robustness through protein fitness (Romero and Arnold, 2009). As a result, higher thermostability can confer greater tolerance to mutations, permitting successful directed evolution strategies (Bloom *et al.*, 2006; Tokuriki and Tawfik, 2009).

**Fig. 1:**
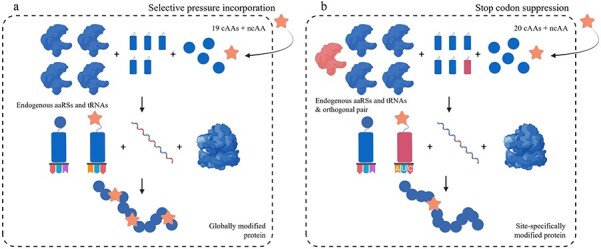
Schematic representation of the methodologies for ncAA incorporation. (**a**) SPI or (**b**) SCS. In blue, endogenous components of the host; in red, the orthogonal pair; ncAA is represented as an orange star, cAAs are represented as blue circles. Figure created using Biorender.com.

For many enzymes, there are no thermostable variants available. To address this problem, several approaches allow the use of the biocatalyst in harsher conditions and formulations, (Haki and Rakshit, 2003) such as (i) identification of novel thermotolerant proteins through genome mining (Zaparucha *et al.*, 2018), (ii) generation of improved well-described enzymes by rational design or random mutagenesis (Wijma *et al.*, 2013, 2018; Shivange *et al.*, 2016; Xu *et al.*, 2020) or (iii) stabilization of proteins through immobilization on matrix materials, such as ceramic surfaces or polymers (Hanefeld *et al.*, 2013; Basso and Serban, 2019). Commonly, these well-described techniques rely on the use of canonical amino acids (cAA), which due to their limited functional group diversity are relatively constrained in their reactivity. This limitation can be overcome through the chemical modification of cAAs to increase the availability of functional groups. The most frequently targeted cAAs are cysteine and lysine due to their high nucleophilicity, with other residues chosen more scarcely (Giri *et al.*, 2021). While site-selective options are available, these methods often give poor control over regio- and chemoselectivity (DeSantis and Jones, 1999; Pagar *et al.*, 2021). Moreover, the required chemistry must be efficient and compatible with aqueous conditions which can be an arduous task (Davis, 2003; Baslé *et al.*, 2010). Conversely, during the last 20 years, robust methods for incorporating non-canonical amino acids (ncAAs) into proteins have emerged, becoming an innovative technology for biotechnology and biocatalysis (Liu and Schultz, 2010). This expansion of the genetic code allowed the addition of a broader set of functional groups to nature’s toolbox, thereby making it an appealing alternative to chemical modification. This permitted chemists to reprogram and exploit the cellular machinery for several purposes, offering new opportunities to improve various enzymatic properties (Lang and Chin, 2014a; Agostini *et al.*, 2017; Drienovska and Roelfes, 2020). Two well-established methods for *in vivo* ncAA incorporation are known, (i) Selective Pressure Incorporation (SPI) and (ii) Stop Codon Suppression (SCS) (Johnson *et al.*, 2010; Dumas *et al.*, 2015). Although both technologies use the cellular translational machinery to produce proteins containing the ncAA of interest, they differ in the degree of protein modification. For the SPI method, the desired ncAAs are usually isostructural to cAA, and therefore globally replace a mimicked residue throughout the whole protein by the endogenous host cell machinery (Fig. 1a). Conversely, SCS allows site-specific incorporation of the respective ncAA using an orthogonal aminoacyl-tRNA synthetase (aaRS)/transfer ribonucleic acid (tRNA) pair as a response to termination codons present at desired positions (Fig. 1b).

**Fig. 2:**
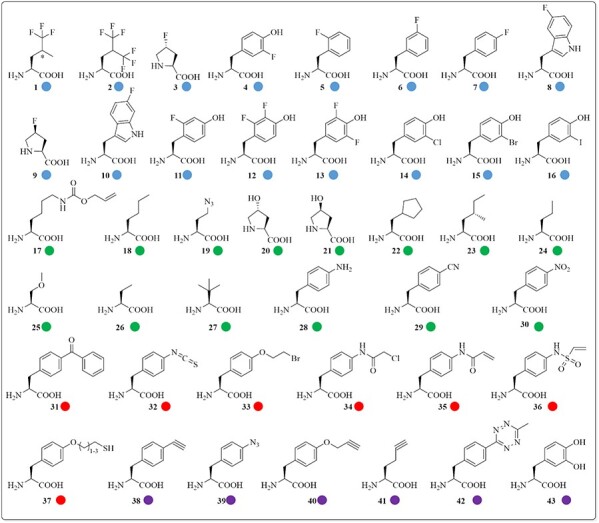
Structures of ncAAs described in this review. The ncAAs are labeled with colors according to each subsection. Blue for halogenated ncAAs, green for non-halogenated ncAAs, red for ncAAs used for crosslinking and purple for ncAAs used for immobilization.

It has been reported that these methods, along with protein engineering or chemical modification, can be used to increase the thermostability of enzymes (Modarres *et al.*, 2016; Rigoldi *et al.*, 2018; Rahban *et al.*, 2022). This review will provide an overview of examples where ncAA incorporation was used for this purpose. For clarity, we will introduce examples of enzymes with globally incorporated ncAAs (using SPI methodology), followed by examples where ncAAs are incorporated specifically at chosen position(s) (using SCS methodology). The sections are concluded with examples of notable exceptions to this classification. The ncAAs referred to throughout this manuscript are summarized in Fig. 2.

## Incorporation of Halogenated ncAAs

Halogenated ncAAs and in particular fluorinated ncAAs have been frequently used to enhance the thermostability of proteins and enzymes. Fluorine is a non-metallic element with the second smallest atomic radius after hydrogen, and due to its high electronegativity exhibits a strong reactivity (Cametti *et al.*, 2012). The unique physicochemical properties of fluorine make it biochemically interesting (Neil and Marsh, 2000). The fluorine bond is similar in size and length to the C–H bond, which allows structural changes with a small chemical footprint, resulting in generally efficient assimilation by the cellular machinery (Cametti *et al.*, 2012; Kumar *et al.*, 2017; Scholfield *et al.*, 2017). In proteins, it has been reported that the introduction of a single fluorine atom in substitution for hydrogen can confer a significant increase in the apparent melting temperature. This effect is due to fluorine atoms maximizing favorable noncovalent interactions with neighboring residues. These interactions are suggested to occur by the so-called σ-hole model (Dunitz, 2004; Politzer and Murray, 2013). The model suggests that in halogen-carbon bonds, the halogen shows an electropositive crown due to a σ-molecular orbital rearrangement, making it amphoteric. This physicochemical property allows it to behave as a halogen donor or hydrogen acceptor, depending on the orientation of the atomic orbitals and on its polarizability.

Early experiments for halogenated ncAA incorporation made use of SPI, and date back to the 1960s with the use of 5′,5′,5′-trifluoroleucine (**1,**Fig. 2). The incorporation of this ncAA was first described in 1963 when it was globally incorporated into *Escherichia coli* proteins (Rennert and Anker, 1963). For this microorganism, at least half of the leucine residues in the bacterial proteins were replaced by **1** almost as readily as leucine itself, presumably due to leucine’s function as protein constituent. Since then, it has been described that the incorporation of **1** can stabilize leucine-zipper peptides, coiled-coil proteins or enzymes such as chloramphenicol acetyltransferase, among others (Tang *et al.*, 2001; Panchenko *et al.*, 2006). In the case of chloramphenicol acetyltransferase from *E. coli* (EcCAT), the residue-specific incorporation of **1** resulted in the conservation of enzymatic activity under ambient temperatures, but a loss in stability against heat and denaturants or organic co-solvents (Panchenko *et al.*, 2006). This could have been caused by inefficient packing of **1** and thereby higher sensitivity to unfolding at increased temperatures. Interestingly, this was ruled out on the basis of a 2-fold increase in secondary structure stabilization after fluorination in EcCAT. Despite this, a large structural destabilization upon increased levels of **1** incorporation occurs at elevated temperatures. For the variant with 80% incorporation, the residual activity was diminished from 83 to 21% after incubating at 60°C, while the half-life was reduced from 160 to 6 min. Hence, the stabilization of the secondary structure does not necessarily lead to an overall increase in thermostability. EcCAT contains seven leucine residues located on the surface, and it was suggested that the presence of fluorine atoms exposed to solvent led to aggregation or misfolding. This detrimental effect was later addressed in a subsequent work by combining the SPI method with directed evolution (Montclare and Tirrell, 2006). After two rounds of random mutagenesis and screening, a triple mutant was selected. The best variant showed a 27-fold improvement in half-life and an increase of 9°C in its temperature of half-inactivation. The obtained variant recovered the loss in thermostability caused by total fluorination and exhibited a modest increase in its specific activity. A structural model showed that each of the mutations was located ˃15 Å away from the binding site, and none make direct contact with any of the trifluoroleucine/leucine residues. While SPI is a powerful technology, it is highly dependent on the cellular machinery of the host (Johnson *et al.*, 2010). This can be observed in the incorporation of hexafluoroleucine (**2**) into the synthetic leucine zipper protein A1, a coiled-coil protein. Although **2** is structurally similar to **1**, successful incorporation required the engineering of a leucyl-tRNA synthetase (Tang and Tirrell, 2001). Nevertheless, incorporation caused a significant improvement in thermal stability by increasing the melting temperature by 22°C and enhancing its chemical tolerance. In some cases, replacing a molecule with a halogenated analog did not lead to an improvement in thermostability, as seen with KlenTaq DNA polymerase (Holzberger and Marx, 2010). Residue-specific replacement of all 32 proline residues located mainly in structural loops by (4*R*)-fluoroproline (**3**) in this polymerase resulted in no clear positive effect.

ω-transaminases (ω-TA) have increasingly been used as efficient biocatalysts due to their ability to produce a wide range of optically pure amine compounds (Mathew and Yun, 2012; Deepankumar *et al.*, 2015). The residue-specific incorporation of *m*-fluorotyrosine (**4**) into ω-TA led to enhanced thermostability (e.g. a residual activity of 36% for ω-TA containing **4** vs 3.3% for the wild-type at 70°C) and dimethyl sulfoxide (DMSO) tolerance without altering substrate specificity and enantioselectivity (Deepankumar *et al.*, 2014). Additionally, the variant exhibited a 2-fold increase in its activity toward acetophenone. Structurally, ω-TA has four tyrosine residues stabilizing its dimer interface, and it is believed that the fluorination of these positions is responsible for the improvement in thermostability. Another biotechnologically relevant example is *Pvu*II, a homodimeric type II restriction endonuclease extensively used in the selective digestion of DNA. The global incorporation of *ortho*-, *meta*- and *para*-fluorophenylalanines (**5–7**) generated various effects (Dominguez Jr *et al.*, 2001). Residue-specific incorporation of **6** resulted in similar conformational stability as the wild-type, with a 2-fold higher specific activity. In contrast, the incorporation of **5** and **7** led to a decrease in both activity and stability. None of the phenylalanine residues in *Pvu*II are close to the DNA recognition or catalytic sites; these contrasting results were conferred to F32. This residue lies at the subunit interface and might play a critical role in the stabilization. However, more experimental data are required to confirm this effect.

S5 phosphotriesterase (PTE) is a homodimer that exhibits a wide substrate scope, including organophosphates and esters (Benning *et al.*, 1994). An initial study showed that the global replacement of five tyrosine residues with **4** improved its thermostability and extended the range of its optimal pH (Votchitseva *et al.*, 2006). The fluorinated variant showed a 3-fold increase in activity at 70°C. The stabilization might occur through additional interactions of the fluorine atoms of the modified Y156 and Y248 with positively charged nitrogen atoms from arginine residues R152 and R246, as well as between the modified Y292 and K294. However, at lower temperatures, the variant exhibited a reduced catalytic efficiency. Y309 is directly involved in the formation of the substrate-binding pocket, and the fluorination at this position might disturb the hydrogen bond distribution. A different work looking at the dimer interface showed that the residue-specific incorporation of *p*-fluorophenylalanine (pFF, **7**) replacing phenylalanine residues, exhibited an increase in stabilization and prevented heat inactivation (Baker and Montclare, 2011). This effect was conferred to the stabilization of hydrophobic interactions along the dimer interface. Kinetically, at room temperature, both the fluorinated variant and the wild-type had similar residual activities. In contrast, at higher temperatures, the wild-type showed a loss of all activity, while the fluorinated variant remained active. Remarkably, the fluorinated variant was able to recover its structure after undergoing thermal unfolding. This effect could be derived from the stabilization across the dimer interface. However, despite the thermal stabilization, the overall fluorinated PTE showed a significant loss in soluble protein yield, suggesting that not all pFF residues were stabilizing. This issue was later addressed by a computational approach, where a Rosetta-based design was used to predict stabilizing site-specific mutations and thermodynamically unfavorable substitutions in pFF-PTE (Yang *et al.*, 2014). In particular, a single modification (F104pFF) located at the interface created energy-demanding clashes with neighboring amino acids. The single mutation to alanine led to a 2-fold higher soluble protein yield than the globally fluorinated pFF-PTE. Furthermore, compared to the wild-type, the pFF-PTE F104A variant showed higher activity at elevated temperatures and maintained residual activity for several days at room temperature.

Budisa *et al*. reported the *in vivo* global incorporation of fluorinated aromatic amino acids analogs (**4**, **7** and 5-fluorotryptophan **8**) in a glycosylation-deficient mutant of *Candida antarctica* lipase B (CalB) (Budisa *et al.*, 2010). Lipases are attractive for the resolution of chiral compounds and the production of biodiesel using transesterification (Ramos-Sánchez *et al.*, 2015). Residue-specific incorporation of **4**, **7** and **8** resulted in prolonged shelf-life of the enzyme activity. The **7**-containing variant showed a 150% higher relative activity at 60°C after several months of storage at 4°C compared to the wild-type enzyme. Conversely, the incorporation of **5**–**7** into tGCN5, a histone acetyltransferase, showed dissimilar results (Voloshchuk *et al.*, 2009). The incorporation of **7** resulted in the least perturbation to the overall structure, whereas the other variants exhibited 6- and 5-fold losses in their half-lives under proteolysis conditions. The destabilizing effects of multiple halogenated ncAAs might reflect steric repulsions arising from increased side-chain volume and/or minor conformational rearrangements for harnessing improved side-chain interactions.

A more challenging approach is to attempt to simultaneously incorporate multiple different ncAAs through SPI (Johnson *et al.*, 2010). For example, three ncAAs were globally incorporated into a lipase from *Thermoanaerobacter thermohydrosulfuricus* (Merkel *et al.*, 2010). All phenylalanine, proline and tryptophan residues were replaced by **7,** (4*S*)-fluoroproline (**9**) and 6-fluorotryptophan (**10**), respectively. The optimum temperature for enzyme activity of the fluorinated enzyme was reduced by 10°C, resulting in a 60% reduction compared to that of the parent protein. Interestingly, despite this extensive 10% modification of the enzyme, it did not cause deleterious structural alterations. This can be explained by monofluorinated amino acids producing fewer structural perturbations, causing them to be more tolerable than bulky ncAAs.

Won and co-workers, developed a more complex cellular system for the *in vivo* biosynthesis of tyrosine analogs catalyzed by a tyrosine phenol-lyase, and their concurrent global incorporation. They tested mono- and disubstituted fluorotyrosines (**4**, **11–13**) in selected enzymes and evaluated their effect on thermostability. The selected enzymes were two ω-TAs from *Sphaerobacter thermophillus* (ω-TAST-I and ω-TAST-II) and an alanine dehydrogenase from *Bacillus subtilis* (AlaDH), enzymes attractive for the synthesis of pharmaceutically important chiral amines (Won *et al.*, 2019). The incorporation of **11** resulted in enhanced thermostability with an increased melting temperature of up to 4°C compared to wild-type enzymes. Furthermore, after incubating at 65°C for 6 h, their residual activity was ~5-fold higher than that of the wild-type enzymes. Interestingly, the incorporation of **11** in the ω-TAST-I and ω-TAST-II resulted in a significant increase in enantiospecificity (*i.e.* increased from 48 to > 99% for TAST-I and 36 to > 99%) for the substrate *S*-methylbenzylamine.

Alternatively, halogenated ncAAs can be incorporated at desired positions using SCS methodology. T4 lysozyme is a model hydrolase with relevant biotechnological applications in medicine, cosmetics, food industry and agriculture (Ferraboschi *et al.*, 2021), and has also been studied for ncAA incorporation by SCS (Carlsson *et al.*, 2018). Mechanistically, for this hydrolase Y18 confers structural stability through water bridges with the nearby E11 and G28. These residues have been found to be critical for both the stability and function of T4 lysozyme (Scholfield *et al.*, 2017; Carlsson *et al.*, 2018). Therefore, for rational ncAA incorporation, *meta-*substituents of tyrosine at position 18 to keep the *p*-hydroxyl moiety intact have been evaluated. Interestingly, among the *m*-halogenated tyrosine analogs (**14**–**16**), only the site-specific incorporation of the chlorinated ncAA (**14**) led to a slightly increased stability and its activity at 40°C. This effect was attributed to the formation of a tight loop between the chlorine and carbonyl oxygen of the peptide bond G28, whereas other halogens did not fit properly (**15** and **16**). The halogen bond potential of chlorine was significantly enhanced due to the polarization via an intramolecular hydrogen bond from the adjacent hydroxyl substituent of the tyrosyl side chain. This resulted in a distinctive synergistic hydrogen bond-enhanced halogen bond interaction.

In another study, the effect of multiple sites incorporation of **14**, **15** or **16** into both glutathione S-transferase (GST) and azoreductase (AZR) was evaluated (Ohtake *et al.*, 2015). For GST, all tyrosine residues throughout the protein were site-specifically replaced. Variants with six halogenated positions incorporating **14** or **15** showed increased values of ΔG_D_(H_2_O) (the difference in Gibbs free energy between unfolded and folded proteins in the absence of denaturant) by 20%. Moreover, these variants showed significantly improved specific activity and heat resistance. Structural analysis showed that the incorporation of these ncAAs generated a tighter protein interior, where the halogen moieties could fill the internal spaces and formed not only van der Waals contacts but also hydrogen bonds and electrostatic interactions. For AZR, seven tyrosine were individually mutated with the brominated ncAAs. After a combinational study of stabilizing single incorporations, a three-site variant was selected. This variant exhibited a 13-fold longer half-life at 78°C and increased ΔG_D_(H_2_O), whereas the wild-type enzyme was nearly inactivated.

Finally, two different codon reassignments can be used to incorporate two different ncAAs within a single enzyme in a site-specific manner. This was observed for a transglutaminase from the actinomycete *Streptoverticillium mobaraense*, where *m*-halogenated tyrosine analogs (**14**–**16**) were incorporated to increase enzyme stability, in addition to *N^ε^*-allyloxycarbonyllysine (AlocKOH) (**17**) to promote protein maturation (Ohtake *et al.*, 2018). The pro-enzyme requires an enzymatic conversion to release an N-terminal inhibitory peptide to obtain the mature enzyme. The incorporation of **14** replacing three tyrosine residues (using stop codon TAG) was obtained after combinational studies of single modifications. This variant exhibited a residual activity 22 times higher and a half-life 5.1-fold longer at 60°C. Additionally, the incorporation of the brominated ncAA (**15**) at these positions achieved a similar degree of heat resistance. While for the iodinated variant (**16**) a decrease in stability was observed. This different effect on stability was conferred to steric hindrance due to the bulkiness of iodine. Structural analysis suggested that the chlorine atom filled a space in the vicinity of the 3′ position of the phenolic ring buried in the protein core, contributing to its increase in thermostability. Subsequently, exploiting the use of an engineered strain, **17** was incorporated (using homoarginine codon AGG) to promote auto-maturation from the pro-enzymes exploiting the α-hydroxy acids of its structure. The resulting self-maturing enzyme showed a 4.7-fold increase in its half-life.

## Incorporation of Non-halogenated ncAAs

In addition to the use of halogenated ncAA to increase the thermostability of enzymes, there are a few reported cases of incorporation of non-halogenated ncAAs for the same purpose (Mendel *et al.*, 1992; Hoesl *et al.*, 2011). Hoesl and co-workers studied the incorporation of ten different isostructural ncAAs in a lipase from *T. thermohydrosulfuricus* (Hoesl *et al*., 2011). For this study, methionine, proline, phenylalanine and tyrosine were replaced via SPI with halogenated (**2**, **4**, **6**, **7**, **9** and **11**) and non-halogenated ncAAs (**18–21**). None of the variants were overall more thermostable than the wild-type, however, three variants retained the optimum temperature along with enhanced activity and a shift in optimal pH (**6**, **11** and **18**). All other variants exhibited a reduction in their optimum temperature in a range of 5–20°C. Furthermore, the norleucine (**18**) incorporating variant, replacing methionine residues, showed a ˃10-fold increase in its activity. Additionally, this variant remained active in the aqueous phase without the need for thermal activation; while the wild-type was nearly inactive without this activation. This effect was suggested to be due to significant global conformational changes, and due to an enhanced hydrophobicity of **18.** The authors suggested that the higher hydrophobicity of the norleucine side chain would be the main reason for better accessibility of the substrate into the enzyme in an aqueous solution. In contrast, in a different work, the global substitution of **18** replacing thirteen methionine residues in cytochrome P450 BM3, while resulting in a nearly 2-fold increased peroxygenase activity, led to reduced thermostability after heat treatment (Cirino *et al.*, 2003).

For site-specific modifications, pioneer work by Peter Schultz and co-workers showed the effect of the incorporation of several ncAAs into T4 lysozyme (**22–27**) (Mendel *et al.*, 1992). The ncAAs replaced a buried leucine to examine their effect on the hydrophobicity, packing, cavity formation and side-chain conformational entropy, in order to influence its protein stability. As expected, the modifications unevenly altered the thermal enzymatic properties. The ncAA incorporation of **22** and **23** resulted in a slight improvement of the thermal stability due to an increase in the bulkiness of buried hydrophobic residues without concomitant strain. In a more recent study, the effect of the incorporation of various ncAAs in a triple mutant of an *E. coli* transketolase was evaluated (Wilkinson and Dalby, 2021). The mutant was found to be less stable than the wild-type, and further exploration of the aromatic ring of Y385 was evaluated due to its critical role in substrate binding. Five different amino acids, including three ncAAs (**28–30**, *p*-aminophenylalanine [*p*-AMF], *p*-cyanophenylalanine [*p-*CNF] and *p*-nitrophenylalanine [*p*-NTF], respectively), as well as tyrosine and phenylalanine were evaluated at this position, aiming to decrease the electron density of the aromatic ring. This approach was suggested to strengthen π–π stacking interactions between position 385, active-site aromatic residues and the substrate. Due to these interactions, active-site rigidity could potentially be increased and thereby the thermal stability. The variant Y385*p-*AMF (**28**) exhibited a slightly higher thermostability and increased activity. This variant exhibited an enhancement in the melting temperature by 3°C, 7.8°C higher than the least stable variant (*p*-NTF) and a 240% improvement in its catalytic efficiency toward 3-hydroxybenzaldehyde. This improvement could be explained by a tight overall packing and increased rigidity of the structure due to the H-bond donor nature of **28***.* Modeling experiments suggested that the stabilization was due to a rearrangement of the hydrogen network forming a helix-turn involving neighboring residues. While the Y385*p-*CNF (**29**) variant showed a slight decrease in the melting temperature by 2°C and a 2-fold increase in the catalytic efficiency. In contrast, Y385*p*-NTF (**30**) showed a decrease in the melting temperature by 4°C and a lower *K_M_* due to its closer proximity to the substrate.

## Crosslinking Strategies Facilitated Through ncAAs

**Fig. 3:**
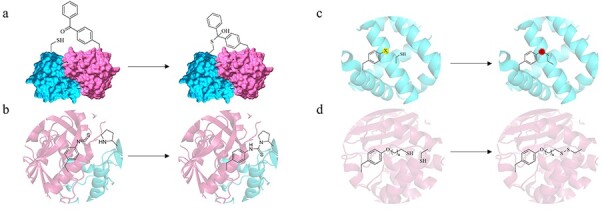
Representation of crosslinking chemistry using ncAAs. Examples of crosslinking using ncAAs are shown. Homoserine O-succinyl transferase dimers (cyan and pink) were stabilized by covalent interactions with (**a**) 31 and a cysteine residue; and (**b**) 32 and an N-terminal proline. (**c**) A set of electrophilic ncAAs (33–36) was evaluated in a myoglobin-based biocatalyst for the covalent interaction with cysteine. (**d**) The interaction of cysteine residues and long-chain thiols (37) was tested on a β-lactamase library of mutants.

Chemical modification methods are highly valuable for increasing enzyme thermostability and other features such as modifying substrate specificity and stereoselectivity (Díaz-Rodríguez and Davis, 2011; Pagar *et al.*, 2021). While several strategies have been developed for most amino acids, traditionally these have been mainly focused on canonical nucleophilic residues (Hacker *et al.*, 2017). Crosslinking using ncAAs represents a new type of (bio)chemistry due to the wide variety of new reactive groups available (Fig. 3). One of the most common examples of ncAA crosslinking makes use of thiol functionalities (Pagar *et al.*, 2021). Thiols exhibit attractive nucleophilicity and can form stabilizing disulfide bridges (Fázio *et al.*, 2006; Liu *et al.*, 2016; Pagar *et al.*, 2021). However, with the expansion of the genetic code, a broader range of ncAAs can be incorporated, including electrophiles (Giri *et al.*, 2021). For example, for homoserine O-succinyl transferase (metA), an essential enzyme from *E. coli* involved in methionine biosynthesis, two different electrophilic ncAAs have been evaluated (**31** and **32**) (Li *et al.*, 2018, 2019). The site-directed incorporation of *p*-benzoylphenylalanine (**31**) increased metA thermostability by crosslinking two monomers (Fig. 3a) (Li *et al.*, 2018). For this transferase, crosslinking improved the melting temperature by 21°C. Structurally, the addition of **31** formed a hemithioketal bond with a nearby cysteine, possibly due to a nucleophilic attack by the cysteine on the carbonyl moiety of benzophenone. In a similar approach, the site-specific incorporation of the electrophilic *p*-isothiocyanate phenylalanine (pNCSF) (**32**) into the surface of metA increased its thermostability by 24°C (Fig. 3b) (Li *et al*., 2019). This effect was reported to be due to the formation of a covalent thiourea bond between the isothiocyanate group from the **32**-side chain with the N-terminal proline of the other monomer. Another successful example is the incorporation of O-(2-bromoethyl) tyrosine (**33**) via SCS on a myoglobin-based cyclopropanation biocatalyst (Moore *et al.*, 2017). A Rosetta-guided design identifying optimal sites for intramolecular crosslinking through redox-stable thioether bonds generated a more stable variant. The prediction led to an enzyme with improved half-inactivation and melting temperatures, by 16 and 18°C, respectively, and greater tolerance to organic solvents. The latter favored more efficient catalysis of a water-insoluble olefin substrate. In a later work, the system was applied using a wider set of electrophilic ncAAs containing chloroacetamido (**34**), acrylamido (**35**) and vinylsulfonamido (**36**) functional groups (Fig. 3c) (Iannuzzelli *et al.*, 2022). The **34**-containing variant showed the most significant improvement in resistance to thermal denaturation with an increase of 27°C and improved temperature-induced heme loss by 30°C. Computational and structural analyses proposed that the **34**-incorporating variant exhibited the fewest energetically feasible conformers and a rigidification of the protein core, which may be an indicator of stability enhancement. Another computational approach using **33** was used for the thermostabilization of multi-modular pullanase (Bi *et al.*, 2021). This led to a variant with a 211% increase in half-life at 70°C and a simultaneous improvement of enzyme activity by 44% compared with wild-type.

A different reported case is the semi-rational design of a library of β-lactamase mutants for the incorporation of thiol harboring ncAAs (Liu *et al.*, 2016). For this chosen model, the aim was to form disulfide bridges between the ncAAs (**37**) and cysteine (Fig. 3d). The library design included random ncAA incorporation and site-defined point mutations for cysteine. This fine-tuning allowed crosslinking between distant cysteine residues, improving the melting temperature by up to 9°C, and maintaining the activity at temperatures that were detrimental to the wild-type. The incorporation of non-canonical thiols produced longer and more flexible disulfide bonds.

## Immobilization of Enzymes Via ncAAs

A well-described alternative for enzyme and protein stabilization is their immobilization on different materials and surfaces (Hanefeld *et al.*, 2013; Basso and Serban, 2019). This approach has led to the successful industrial application of a wide variety of enzymes (Hanefeld *et al.*, 2013). Currently, there are several ways to immobilize proteins, such as mechanical entrapment, ionic adsorption and covalent interactions, among others (Basso and Serban, 2019; Heckmann and Paradisi, 2020). However, an ideal system should decrease the high unpredictability of the complex matrix. After immobilization, the enzyme activity may be reduced. This occurs by non-optimal orientation, which results in blockage of the active site and/or binding pocket or by a simple loss of motility (Hernandez and Fernandez-Lafuente, 2011; Hanefeld *et al.*, 2013; Basso and Serban, 2019; Pagolu *et al.*, 2021). To address this issue, strategies for site-specific enzyme immobilization have emerged in recent years. Through molecular biology techniques, new approaches for covalent bonding with a desired material surface are available by varying the position of the mutation (Hernandez and Fernandez-Lafuente, 2011; Heckmann and Paradisi, 2020).

**Fig. 4:**
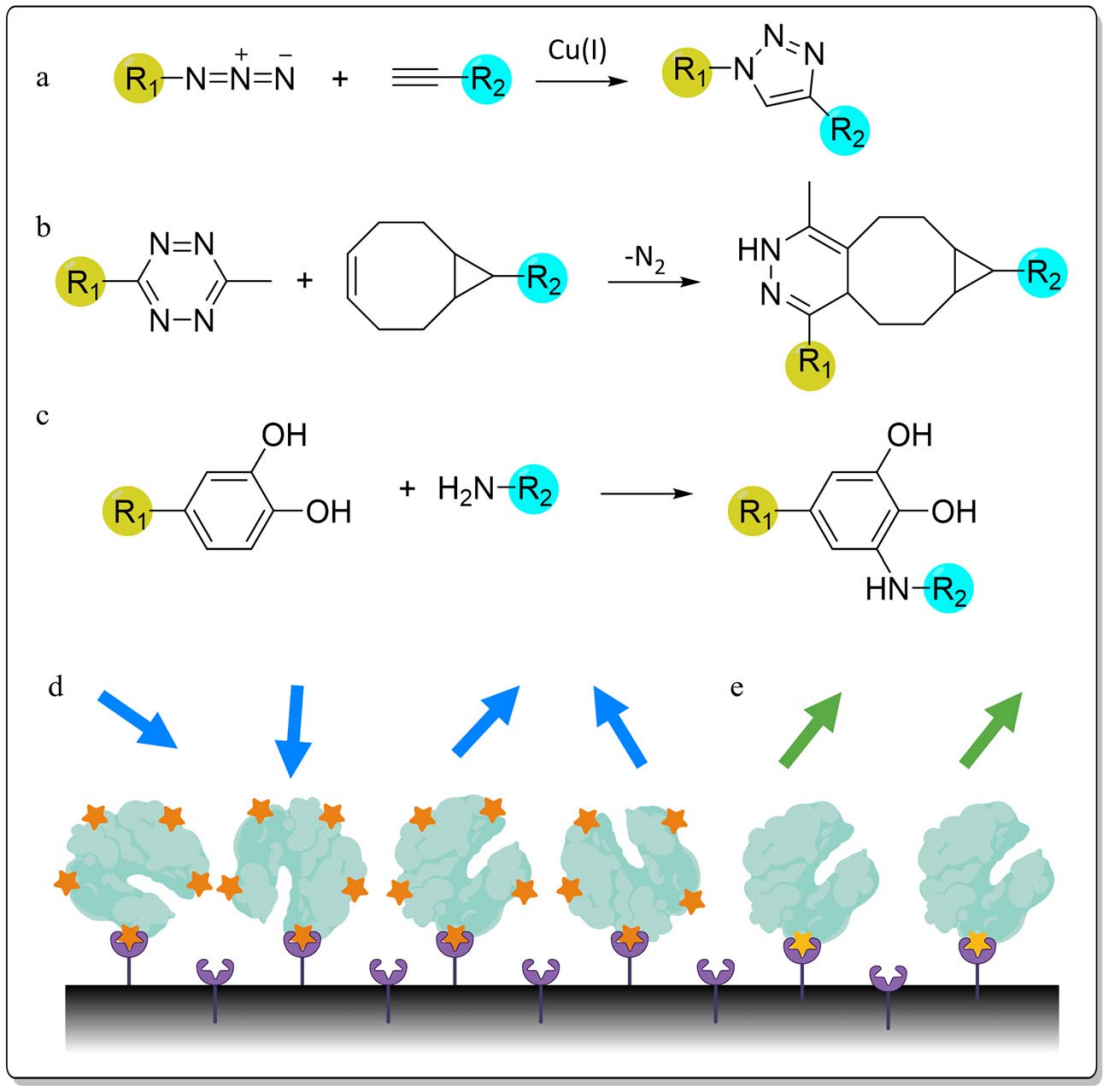
Immobilization methods using ncAAs. Examples of click-chemistry reactions for protein immobilization: (**a**) Huisgen 1,3-dipolar cycloaddition, (**b**) inverse-electron-demand Diels-Alder reaction and (**c**) 43-mediated immobilization. R1 and R2 represent the target protein or functionalized surface for immobilization. (**d, e**) Schematic representation of the immobilization strategies through ncAAs: (d) immobilization of proteins globally incorporating ncAAs and (e) site-oriented immobilization. The colored arrows represent the orientation and exposition of the active site. The ncAAs are represented as orange or yellow stars. Figure created with BioRender.com.

The Cu(I)-catalyzed azide-alkyne cycloaddition or Huisgen 1,3-dipolar cycloaddition has been widely reported as an efficient reaction by binding an azido and an alkynyl group in the presence of a copper catalyst (Fig. 4a) (Meldal and Tornøe, 2008). Through this copper-catalyzed reaction, CalB was attached to alkyne-functionalized PEG(5000) after global incorporation of azidohomoalanine (**19**) by SPI (Fig. 4d) (Schoffelen *et al.*, 2008). Five methionine residues were replaced by **19**, four of the azide-containing ncAAs were buried in the core of CalB, and one residue was solvent exposed. This localization resulted in only one ncAA side chain accessible for chemical modification, producing a quasi-monofunctionalized enzyme. While the immobilization was successful, the activity was slightly decreased. In a different study, this azide-modified CalB was attached to polymersome surfaces through the same click reaction (van Dongen *et al.*, 2008). The azido-functionalized enzyme retained its activity while immobilized on the material.

As described above, SCS technology allows site-specific incorporation of new functional groups. This quality is especially attractive for site-oriented immobilization (Fig. 4e). This can be observed for the immobilization of murine dihydrofolate reductase, an enzyme involved in tetrahydrofolate biosynthesis, on streptavidin-coated plates using *p*-ethynylphenylalanine (**38**) (Lim *et al.*, 2014). This ncAA was incorporated into positions with high and low exposure to solvent. The obtained variants were immobilized by reacting the alkyne group with biotin-PEG3-azide and were shown to retain their catalytic activity. Similarly, for an aldehyde ketone reductase, five residues were each replaced by *p*-azidophenylalanine (**39**) to immobilize the enzyme on an epoxy resin using the same click reaction (Li *et al.*, 2020). The five-point immobilized enzyme exhibited an improved half-life 13 and 7 times higher than that of the free enzyme at 30 and 60°C, respectively. This effect was obtained due to precise chemical attachment protecting the active site during covalent immobilization. In a similar approach, T4 lysozyme was immobilized by the Huisgen 1,3-dipolar cycloaddition reaction on azide functionalized beads (Wu *et al.*, 2015). For this reaction, *p*-propargyloxyphenylalanine (**40**) was site-directedly incorporated at various positions with high surface accessibility. Two of the site-oriented immobilized enzymes showed an increase in their activity, and a higher level of recovery after freeze–thaw cycles and under urea incubation than the randomly immobilized enzyme and the wild-type. *Gaussia* luciferase is an enzyme that catalyzes the oxidation of coelenterazine producing an intense blue light with a short emission half-life (Welsh *et al.*, 2009). The enzyme was attached to PEG after the global incorporation of **19** or homopropargylglycine (**41**) replacing methionine in a cell-free manner. Through the copper-click reaction, the immobilized variants exhibited a 3-fold increase in luminescence half-life, while retaining wild-type luminescence intensity.

An alternative to the azide-alkyne cycloaddition is to immobilize proteins by inverse-electron-demand Diels-Alder reactions (Fig. 4b). This reaction can be performed between tetrazines and strained *trans*-cyclooctenes exhibiting high rates and decreased non-specific adsorption (Blackman *et al.*, 2008; Lang and Chin, 2014b). An example of this attractive reaction is the immobilization of the human carbonic anhydrase II (CA2), which catalyzes the reversible hydration of carbon dioxide (Bednar *et al.*, 2019). The incorporation of tetrazine (**42**) in residues exposed to solvent, either parallel to the surface or aimed toward the surface, allowed global immobilization of at least 90%. The site-specifically oriented CA2 variants retained activities between 60 and 90%. This reaction has also been reported for protein immobilization in an orientationally defined manner through the incorporation of a modified tyrosine-based ncAA with a tetrazine moiety (Bednar *et al.*, 2019).

Finally, a double approach has been described using ω-TA through coupling the residue- and site-specific incorporation strategies (Deepankumar *et al.*, 2015). In this study, **3** was globally incorporated, while 3,4-dihydroxyphenylalanine (**43**) was incorporated site-specifically. Initially, site-directed incorporation of **43** was shown to increase the thermostability of green fluorescent protein by acting as a protein crosslinking agent upon periodate oxidation (Fig. 4c) (Burdine *et al.*, 2004; Deepankumar *et al.*, 2017). Furthermore, the site-oriented incorporation of **43** into ω-TA allowed successful immobilization of the variant on chitosan and PEG amine-containing polystyrene beads with high reusability (up to 10 cycles) (Deepankumar *et al.*, 2015). The double ncAA incorporating variant (with **3** and **43**) showed a 2-fold enhancement in the half-lives at 50, 60 and 70°C. Additionally, the immobilized ω-TA variant maintained high enantioselectivity and showed increased conversion levels compared to the wild-type. Several other ncAAs have been evaluated for protein immobilization and labeling, such as homoallylglycine, azidonorleucine and acetylphenylalanine, among others. Although they have interesting prospects, they have not or scarcely been evaluated in enzymes to date (Lang and Chin, 2014b; Agostini *et al.*, 2017; Deepankumar *et al.*, 2017; Saleh *et al.*, 2019).

## Conclusion & Outlook

Biocatalysis is an exciting field where disciplines such as biochemistry, molecular biology and organic chemistry are constantly combined. The field is maturing to become a green alternative to classical chemical transformations. Consequently, major progress has been made in improving different parameters where enzymes show disadvantages compared to chemical methods, such as their low thermostability. To address this problem, various strategies have been studied, including computational design, directed evolution and genome mining, among others (Akbulut *et al.*, 2013; Satyanarayana *et al.*, 2013; Wijma *et al.*, 2013, 2018; Rigoldi *et al.*, 2018). In this work, we reviewed the research to date on enzyme thermal stabilization by ncAA incorporation. These strategies are biotechnologically attractive for several reasons. Unlike chemical modification strategies, ncAA incorporation by SPI or SCS does not require further enzyme purification or steps since it occurs within the host. Moreover, although chemical modification with cAAs has been successfully performed, most methods are still based on reactions with limited scope, selectivity and efficiency, often resulting in heterogeneous mixtures (Pagar *et al.*, 2021). In addition, ncAA incorporation allows further optimization of enzymes through powerful methods, such as directed evolution (Mayer *et al.*, 2019). Compared with classical enzyme engineering methods, there are relevant examples (besides the ones described in this review) where major mutagenesis campaigns using cAAs have failed, but rational ncAA incorporation improved desired enzymatic parameters, such as stability or even activity (Ugwumba *et al.*, 2011). Although it remains to be fully understood, there is no doubt that the use of (bio)synthetic ncAAs provides access to new and valuable functional chemistry for enzyme engineering.

The different approaches toward enzyme stabilization by ncAA incorporation that have been discussed in this review suggest to us that we are only scratching the surface regarding the potential they have to offer. For the last decades, the use of halogenated amino acids for the thermal stabilization of enzymes was a frequent alternative, and some general relevant outcomes can be considered from these studies. Strategies like halogenated ncAA incorporation into dimer–dimer interfaces or localization into the enzyme core to form synergistic interplay between the hydrogen bonds and halogen moieties can be considered. Unfortunately, there is no single straightforward method to improve enzyme thermostability using ncAAs. In addition, modifications in the structure of ncAAs can also affect enzyme function. The impact of these changes is specific to both the ncAA and the protein it interacts with, since factors such as the chemical properties of the ncAA (such as hydrophobicity, bulkiness or nucleophilicity) affect the protein differently depending on its microenvironment. It must be considered, that while new side chains can be incorporated aiming to increase stability, structural changes can rearrange interactions within the enzyme, affecting and perturbing the distribution of the network of interactions for the catalysis. Similar to classical enzyme engineering, the rational design of ncAA incorporation through computational methods, considering relevant structural and chemical knowledge, is a powerful tool to successfully design robust enzymes (Merkel *et al.*, 2010; Moore *et al.*, 2017; Carlsson *et al.*, 2018; Bi *et al.*, 2021). The use of these methods can not only lead to the design of more stable enzymes, but also reduce the need for extensive experimental efforts to achieve this goal. Additionally, thermostabilization through crosslinking and immobilization exploiting the chemistry of ncAAs have been reported as attractive strategies. Careful site-oriented immobilization control can minimize the effects of steric hindrance of the enzyme active site and provide a greater understanding of enzyme-surface interactions (Hernandez and Fernandez-Lafuente, 2011; Basso and Serban, 2019; Bednar *et al.*, 2019). Further investigation on chemical reactivity using ncAAs can make these systems even more attractive through new chemistries.

Despite many hurdles yet to be overcome, a pressing concern in the field is the limited accessibility of ncAAs and the systems for their incorporation. The high cost of ncAAs has been a significant obstacle, but there have been promising advances in addressing this issue, such as developing novel and cheap chemical synthetic routes or *in vivo* biosynthetic pathways for the production of ncAAs using cellular machinery (Chen *et al.*, 2018; Kim *et al.*, 2018; Won *et al.*, 2019). While there is constant development to tackle critical challenges, more multidisciplinary efforts need to be made. To address these issues, an interplay between the fields of synthetic biology, organic chemistry, computational biology and biochemistry is required. Collaborative campaigns will make biotechnological and biocatalytic approaches more efficient and reliable, making biocatalysis an even more appealing alternative to classical chemical methods.

## Conflict of interest

The authors declare no conflict of interest.

## Funding

The authors acknowledge financial support from the Netherlands Organization for Scientific Research (NWO VI.Veni.202.166).

## References

[ref1] Agostini, F., Völler, J., Koksch, B. et al. (2017) Angew. Chem. Int. Ed., 129, 9810–9835.

[ref2] Akbulut, N., Öztürk, M.T., Pijning, T. et al. (2013) J. Biotechnol., 164, 123–129. 23313890 10.1016/j.jbiotec.2012.12.016

[ref3] Baker, P.J. and Montclare, J.K. (2011) ChemBioChem, 12, 1845–1848. 21710682 10.1002/cbic.201100221

[ref4] Baslé, E., Joubert, N. and Pucheault, M. (2010) Chem. Biol., 17, 213–227. 20338513 10.1016/j.chembiol.2010.02.008

[ref5] Basso, A. and Serban, S. (2019) Mol. Catal., 479, 110607.

[ref6] Bednar, R.M., Golbek, T.W., Kean, K.M. et al. (2019) ACS Appl. Mater. Interfaces, 11, 36391–36398. 31525993 10.1021/acsami.9b12746

[ref7] Bell, E.L., Finnigan, W., France, S.P. et al. (2021) Nat. Rev. Dis. Primers., 1, 1–21.

[ref8] Benning, M.M., Kuo, J.M., Raushel, F.M. et al. (1994) Biochem., 33, 15001–15007. 7999757 10.1021/bi00254a008

[ref9] Bi, J., Jing, X., Wu, L. et al. (2021) Comput. Struct. Biotechnol. J., 19, 577–585. 33510863 10.1016/j.csbj.2020.12.043 PMC7811066

[ref10] Blackman, M.L., Royzen, M. and Fox, J.M. (2008) J. Am. Chem. Soc., 130, 13518–13519. 18798613 10.1021/ja8053805 PMC2653060

[ref11] Bloom, J.D., Labthavikul, S.T., Otey, C.R. et al. (2006) PNAS, 103, 5869–5874. 16581913 10.1073/pnas.0510098103 PMC1458665

[ref12] Bommarius, A.S. and Paye, M.F. (2013) Chem. Soc. Rev., 42, 6534–6565. 23807146 10.1039/c3cs60137d

[ref13] Budisa, N., Wenger, W. and Wiltschi, B. (2010) Mol. Biosyst., 6, 1630–1639. 20431819 10.1039/c002256j

[ref14] Burdine, L., Gillette, T.G., Lin, H.J. et al. (2004) J. Am. Chem. Soc., 126, 11442–11443. 15366882 10.1021/ja045982c

[ref15] Cametti, M., Crousse, B., Metrangolo, P. et al. (2012) Chem. Soc. Rev., 41, 31–42. 21691620 10.1039/c1cs15084g

[ref16] Carlsson, A.C.C., Scholfield, M.R., Rowe, R.K. et al. (2018) Biochemistry, 57, 4135–4147. 29921126 10.1021/acs.biochem.8b00603 PMC6052408

[ref17] Chen, Y., Loredo, A., Gordon, A. et al. (2018) Chem. Commun., 54, 7187–7190. 10.1039/c8cc03819h 29896591

[ref18] Cirino, P.C., Tang, Y., Takahashi, K. et al. (2003) Biotechnol. Bioeng., 83, 729–734. 12889037 10.1002/bit.10718

[ref19] Davis, B.G. (2003) Curr. Opin. Biotechnol., 14, 379–386. 12943846 10.1016/s0958-1669(03)00098-3

[ref20] Deepankumar, K., Nadarajan, S.P. et al. (2015) ChemCatChem, 7, 417–421.

[ref21] Deepankumar, K., Prabhu, N.S., Kim, J.H. et al. (2017) Biotechnol Bioprocess Eng, 22, 248–255.

[ref22] Deepankumar, K., Shon, M., Nadarajan, S.P. et al. (2014) Adv Synth Catal, 356, 993–998.

[ref23] DeSantis, G. and Jones, J.B. (1999) Curr. Opin. Biotechnol., 10, 324–330. 10449313 10.1016/S0958-1669(99)80059-7

[ref24] Díaz-Rodríguez, A. and Davis, B.G. (2011) Curr. Opin. Chem. Biol., 15, 211–219. 21276746 10.1016/j.cbpa.2010.12.002

[ref25] Dominguez, M.A. Jr., Thornton, K.C., Melendez, M.G. et al. (2001) PEDS, 45, 55–61. 10.1002/prot.1123 11536360

[ref26] van Dongen, S.F.M., Nallani, M., Schoffelen, S. et al. (2008) Macromol. Rapid Commun., 29, 321–325.

[ref27] Drienovska, I. and Roelfes, G. (2020) Nat. Catal., 3, 193–202.

[ref28] Dumas, A., Lercher, L., Spicer, C.D. et al. (2015) Chem. Sci., 6, 50–69. 28553457 10.1039/c4sc01534g PMC5424465

[ref29] Dunitz, J.D. (2004) Chem Bio Chem, 5, 614–621.

[ref30] Fázio, M.A., Oliveira, V.X. Jr., Bulet, P. et al. (2006) J. Pept. Sci., 84, 205–218. 10.1002/bip.20396 16235231

[ref31] Ferraboschi, P., Ciceri, S. and Grisenti, P. (2021) Antibiotics, 10, 1534. 34943746 10.3390/antibiotics10121534 PMC8698798

[ref32] Fürst, M.J.L.J., Gran-Scheuch, A., Aalbers, F.S. et al. (2019) ACS Catal., 9, 11207–11241.

[ref33] Giri, P., Pagar, A.D., Patil, M.D. et al. (2021) Biotechnol. Adv., 53, 107868. 34774927 10.1016/j.biotechadv.2021.107868

[ref34] Hacker, S.M., Backus, K.M., Lazear, M.R. et al. (2017) Nat. Chem., 9, 1181–1190. 29168484 10.1038/nchem.2826 PMC5726523

[ref35] Haki, G.D. and Rakshit, S.K. (2003) Bioresour. Technol., 89, 17–34. 12676497 10.1016/s0960-8524(03)00033-6

[ref36] Han, T., Zeng, F., Li, Z. et al. (2013) Lett. Appl. Microbiol., 57, 336–343. 23789737 10.1111/lam.12118

[ref37] Hanefeld, U., Cao, L. and Magner, E. (2013) Chem. Soc. Rev., 42, 6211–6212. 23807229 10.1039/c3cs90042h

[ref38] Hanefeld, U., Hollmann, F. and Paul, C.E. (2022) Chem. Soc. Rev., 51, 594–627. 34929722 10.1039/d1cs00100k

[ref39] Heckmann, C.M. and Paradisi, F. (2020) ChemCatChem, 12, 6082–6102. 33381242 10.1002/cctc.202001107 PMC7756376

[ref40] Hernandez, K. and Fernandez-Lafuente, R. (2011) Enzyme Microb. Technol., 48, 107–122. 22112819 10.1016/j.enzmictec.2010.10.003

[ref41] Hoesl, M.G., Acevedo-Rocha, C.G., Nehring, S. et al. (2011) ChemCatChem, 3, 213–221.

[ref42] Holzberger, B. and Marx, A. (2010) J. Am. Chem. Soc., 132, 15708–15713. 20961065 10.1021/ja106525y

[ref43] Iannuzzelli, J.A., Bacik, J.P., Moore, E.J. et al. (2022) Biochemistry, 61, 1041–1054. 35612958 10.1021/acs.biochem.2c00033 PMC9178789

[ref44] Johnson, J.A., Lu, Y.Y., van Deventer, J.A. et al. (2010) Curr. Opin. Chem. Biol., 14, 774–780. 21071259 10.1016/j.cbpa.2010.09.013 PMC3008400

[ref45] Kim, S., Sung, B.H., Kim, S.C. et al. (2018) Chem. Commun., 54, 3002–3005. 10.1039/c8cc00281a 29508865

[ref46] Koch, R., Zablowski, P., Spreinat, A. et al. (1990) FEMS Microbiol. Lett., 71, 21–26.

[ref47] Kumar, V., Mulder, D.J., Cavallo, G. et al. (2017) J. Fluor. Chem., 198, 54–60.

[ref48] Lang, K. and Chin, J.W. (2014a) Chem. Rev., 114, 4764–4806. 24655057 10.1021/cr400355w

[ref49] Lang, K. and Chin, J.W. (2014b) ACS Chem. Biol., 9, 16–20. 24432752 10.1021/cb4009292

[ref50] Li, H., Yin, Y., Wang, A. et al. (2020) RSC Adv., 10, 2624–2633. 35496112 10.1039/c9ra09067c PMC9049136

[ref51] Li, J.C., Liu, T., Wang, Y. et al. (2018) J. Am. Chem. Soc., 140, 15997–16000. 30433771 10.1021/jacs.8b07157 PMC6426444

[ref52] Li, J.C., Nastertorabi, F., Xuan, W. et al. (2019) ACS Chem. Biol., 14, 1150–1153. 31181898 10.1021/acschembio.9b00002 PMC6791372

[ref53] Lim, S.I., Mizuta, Y., Takasu, A. et al. (2014) PloS One, 9, e98403. 24887377 10.1371/journal.pone.0098403 PMC4041766

[ref54] Liu, C.C. and Schultz, P.G. (2010) Annu. Rev. Biochem., 79, 413–444. 20307192 10.1146/annurev.biochem.052308.105824

[ref55] Liu, T., Wang, Y., Luo, X. et al. (2016) PNAS, 113, 5910–5915. 27162342 10.1073/pnas.1605363113 PMC4889405

[ref56] Mathew, S. and Yun, H. (2012) ACS Catal., 2, 993–1001.

[ref57] Mayer, C., Dulson, C., Reddem, E. et al. (2019) Angew. Chem. Int. Ed., 58, 2083–2087. 10.1002/anie.201813499 PMC6519144 30575260

[ref58] Meldal, M. and Tornøe, C.W. (2008) Chem. Rev., 108, 2952–3015. 18698735 10.1021/cr0783479

[ref59] Mendel, D., Ellman, J.A., Chang, Z. et al. (1992) Science, 256, 1798–1802. 1615324 10.1126/science.1615324

[ref60] Merkel, L., Schauer, M., Antranikian, G. et al. (2010) ChemBioChem, 11, 1505–1507. 20572253 10.1002/cbic.201000295

[ref61] Modarres, H.P., Mofrad, M.R. and Sanati-Nezhad, A. (2016) RSC Adv., 6, 115252–115270.

[ref62] Montclare, J.K. and Tirrell, D.A. (2006) Angew. Chem. Int. Ed., 118, 4630–4633.

[ref63] Moore, E.J., Zorine, D., Hansen, W.A. et al. (2017) PNAS, 114, 12472–12477. 29109284 10.1073/pnas.1708907114 PMC5703291

[ref64] Neil, E. and Marsh, G. (2000) Chem. Biol., 7, R153–R157. 10903940 10.1016/s1074-5521(00)00139-3

[ref65] Ohtake, K., Mukai, T., Iraha, F. et al. (2018) ACS Synth. Biol., 7, 2170–2176. 30063837 10.1021/acssynbio.8b00157

[ref66] Ohtake, K., Yamaguchi, A., Mukai, T. et al. (2015) Sci. Rep., 5, 1–6. 10.1038/srep09699 PMC4434889 25982672

[ref67] Pagar, A.D., Patil, M.D., Flood, D.T. et al. (2021) Chem. Rev., 121, 6173–6245. 33886302 10.1021/acs.chemrev.0c01201

[ref68] Pagolu, R., Singh, R., Shanmugam, R. et al. (2021) Bioresour. Technol., 331, 125063. 33813167 10.1016/j.biortech.2021.125063

[ref69] Panchenko, T., Zhu, W.W. and Montclare, J.K. (2006) Biotechnol. Bioeng., 94, 921–930. 16548001 10.1002/bit.20910

[ref70] Politzer, P. and Murray, J.S. (2013) Chem Phys Chem, 14, 278–294. 23303575 10.1002/cphc.201200799

[ref71] Rahban, M., Zolghadri, S., Salehi, N. et al. (2022) Int. J. Biol. Macromol., 214, 642–654. 35772638 10.1016/j.ijbiomac.2022.06.154

[ref72] Ramos-Sánchez, L.B., Cujilema-Quitio, M.C., Julian-Ricardo, M.C. et al. (2015) J Bioprocess Biotech., 5, 1–9.

[ref73] Rennert, O.M. and Anker, H.S. (1963) Biochemistry, 2, 471–476. 14069531 10.1021/bi00903a013

[ref74] Rigoldi, F., Donini, S., Redaelli, A. et al. (2018) APL Bioeng., 2, 011501. 31069285 10.1063/1.4997367 PMC6481699

[ref75] Romero, P.A. and Arnold, F.H. (2009) Nat. Rev. Mol. Cell Biol., 10, 866–876. 19935669 10.1038/nrm2805 PMC2997618

[ref76] Saleh, A.M., Wilding, K.M., Calve, S. et al. (2019) J. Biol. Eng., 13, 1–14. 31139251 10.1186/s13036-019-0166-3 PMC6529998

[ref77] Sarmiento, F., Peralta, R. and Blamey, J.M. (2015) Front. Bioeng. Biotechnol., 3, 148. 26539430 10.3389/fbioe.2015.00148 PMC4611823

[ref78] Satyanarayana, T., Littlechild, J. and Kawarabayasi, Y. (2013) Biotechnol Thermophiles, 3.

[ref79] Schoffelen, S., Lambermon, M.H.L., van Eldijk, M.B. et al. (2008) Bioconjug. Chem., 19, 1127–1131. 18461981 10.1021/bc800019v

[ref80] Scholfield, M.R., Ford, M.C., Carlsson, A.C.C. et al. (2017) Biochemistry (Wash), 56, 2794–2802. 28345933 10.1021/acs.biochem.7b00022

[ref81] Sharma, A. and Satyanarayana, T. (2012) Extremophiles, 16, 515–522. 22527045 10.1007/s00792-012-0451-2

[ref82] Sheldon, R.A. and Woodley, J.M. (2018) Chem. Rev., 118, 801–838. 28876904 10.1021/acs.chemrev.7b00203

[ref83] Shivange, A.V., Roccatano, D. and Schwaneberg, U. (2016) Appl. Microbiol. Biotechnol., 100, 227–242. 26403922 10.1007/s00253-015-6959-5

[ref84] Tang, Y., Ghirlanda, G., Petka, W.A. et al. (2001) Angew. Chem. Int. Ed., 40, 1494–1496. 11317312

[ref85] Tang, Y. and Tirrell, D.A. (2001) J. Am. Chem. Soc., 123, 11089–11090. 11686725 10.1021/ja016652k

[ref86] Tokuriki, N. and Tawfik, D.S. (2009) Curr. Opin. Struct. Biol., 19, 596–604. 19765975 10.1016/j.sbi.2009.08.003

[ref87] Ugwumba, I.N., Ozawa, K., Xu, Z.Q. et al. (2011) J. Am. Chem. Soc., 133, 326–333. 21162578 10.1021/ja106416g

[ref88] Voloshchuk, N., Zhu, A.Y., Snydacker, D. et al. (2009) Bioorg. Med. Chem. Lett., 19, 5449–5451. 19666222 10.1016/j.bmcl.2009.07.093

[ref89] Votchitseva, Y.A., Efremenko, E.N. and Varfolomeyev, S.D. (2006) Russ. Chem Bull., 55, 369–374.

[ref90] Welsh, J.P., Patel, K.G., Manthiram, K. et al. (2009) Biochem. Biophys. Res. Commun., 389, 563–568. 19825431 10.1016/j.bbrc.2009.09.006

[ref91] Wijma, H.J., Floor, R.J. and Janssen, D.B. (2013) Curr. Opin. Struct. Biol., 23, 588–594. 23683520 10.1016/j.sbi.2013.04.008

[ref92] Wijma, H.J., Fürst, M.J.L.J. and Janssen, D.B. (2018) Methods Mol. Biol., 1685, 69–85. 29086304 10.1007/978-1-4939-7366-8_5

[ref93] Wilkinson, H.C. and Dalby, P.A. (2021) FEBS J., 288, 1935–1955. 32897608 10.1111/febs.15560

[ref94] Winkler, C.K., Schrittwieser, J.H. and Kroutil, W. (2021) ACS Cent. Sci., 7, 55–71. 33532569 10.1021/acscentsci.0c01496 PMC7844857

[ref95] Won, Y., Jeon, H., Pagar, A.D. et al. (2019) Chem. Commun., 55, 15133–15136. 10.1039/c9cc08503c 31789331

[ref96] Wu, J.C.Y., Hutchings, C.H., Lindsay, M.J. et al. (2015) J. Biotechnol., 193, 83–90. 25449015 10.1016/j.jbiotec.2014.10.039

[ref97] Xu, Z., Cen, Y.K., Zou, S.P. et al. (2020) Crit. Rev. Biotechnol., 40, 83–98. 31690132 10.1080/07388551.2019.1682963

[ref98] Yang, C., Renfrew, P.D., Olsen, A.J. et al. (2014) ChemBioChem, 15, 1761–1764. 25066940 10.1002/cbic.201402062

[ref99] Yi, D., Bayer, T., Badenhorst, C.P.S. et al. (2021) Chem. Soc. Rev., 50, 8003–8049. 34142684 10.1039/d0cs01575j PMC8288269

[ref100] Zaparucha, A., de Berardinis, V. and Vaxelaire-Vergne, C. (2018) Royal Soc. Chem., 1–27.

